# Myelopoiesis of the Amphibian *Xenopus laevis* Is Segregated to the Bone Marrow, Away From Their Hematopoietic Peripheral Liver

**DOI:** 10.3389/fimmu.2019.03015

**Published:** 2020-01-22

**Authors:** Amulya Yaparla, Phillip Reeves, Leon Grayfer

**Affiliations:** ^1^Department of Biological Sciences, George Washington University, Washington, DC, United States; ^2^School Without Walls High School, Washington, DC, United States

**Keywords:** hematopoiesis, myelopoiesis, peripheral liver, bone marrow, CXCL12

## Abstract

Across vertebrates, hematopoiesis takes place within designated tissues, wherein committed myeloid progenitors further differentiate toward cells with megakaryocyte/erythroid potential (MEP) or those with granulocyte/macrophage potential (GMP). While the liver periphery (LP) of the *Xenopus laevis* amphibian functions as a principal site of hematopoiesis and contains MEPs, cells with GMP potential are instead segregated to the bone marrow (BM) of this animal. Presently, using gene expression and western blot analyses of blood cell lineage-specific transcription factors, we confirmed that while the *X. laevis* LP hosts hematopoietic stem cells and MEPs, their BM contains GMPs. In support of our hypothesis that cells bearing GMP potential originate from the frog LP and migrate through blood circulation to the BM in response to chemical cues; we demonstrated that medium conditioned by the *X. laevis* BM chemoattracts LP and peripheral blood cells. Compared to LP and by examining a comprehensive panel of chemokine genes, we showed that the *X. laevis* BM possessed greater expression of a single chemokine, CXCL12, the recombinant form of which was chemotactic to LP and peripheral blood cells and appeared to be a major chemotactic component within BM-conditioned medium. In confirmation of the hepatic origin of the cells that give rise to these frogs' GMPs, we also demonstrated that the *X. laevis* BM supported the growth of their LP-derived cells.

## Introduction

Across all vertebrates, pluripotent pre-committed and lineage-committed blood cell precursors reside within designated hematopoietic sites (1). During blood cell lineage commitment, the pluripotent stem cells give rise to common lymphoid (2) and common myeloid (3) progenitors (CLPs and CMPs, respectively). The CLPs further differentiate into B and T cell precursors, while the CMPs give rise to precursors of the megakaryocyte/erythroid (MEPs) or granulocyte/macrophage potential (GMPs) (3). Site(s) of adult animal hematopoiesis vary across vertebrata, from bone marrow in reptiles (4), birds (5, 6), and most mammalian species (7) to the head kidney in teleost fish (8, 9). In turn, the differentiation of GMPs toward the macrophage lineage depends on the activation of the colony stimulating factor-1 receptor (CSF-1R) (10) by its cognate ligand, colony stimulating factor-1 (CSF-1), which acts as a monopoietic growth factor (11, 12). Similarly, the differentiation of MEPs toward the erythroid lineage depends on erythropoietin (EPO) (13, 14). Notably, while the peripheral (subcapsular) liver of the anuran amphibian *Xenopus laevis* is considered to be the principal site of hematopoiesis (14–16), we demonstrated that the cells responsive to CSF-1 reside in the *X. laevis* bone marrow and are absent from their peripheral liver (17, 18). Conversely, we (17) and others (14) showed that the *X. laevis* peripheral liver, but not their bone marrow, contains cells that respond to EPO to form erythroid-lineage cells. To date, the ontogeny of *X. laevis* bone marrow GMPs remains poorly understood.

The step-wise lineage commitment of pluripotent cells depends on external stimuli, including specific growth factors akin to CSF-1 and EPO, and progenitor cell-stromal cell interactions (19, 20). Concurrently, these commitment steps are marked by changes in gene expression of cell lineage-specific transcription factors, which are thus often used as markers to identifying the respective, lineage-committed cell populations (19–21). For example and pertinently to this work, as CMPs commit to MEPs or GMPs, they exhibit increased expression of *fli1, gata1*, and *nfe2* or *pu1, egr1, egr2*, and *gfi1*, respectively (19–21).

The retention of progenitors and certain committed blood cells within hematopoietic tissues, as well as the mobilization and homing of specific cell populations to disparate tissues within organisms, is mediated by designated chemokines (22). In general, chemokines are classified into four families based on the presence and positioning of conserved cysteine residues: C, CC, CXC, and CX3C (23). Chemokines typically act through cell-surface G-protein-coupled seven-transmembrane receptors and have been most thoroughly described in the context of leukocyte recruitment during immune responses (23). Conversely, the roles of chemokines in hematopoiesis first became evident from the analyses of the interaction between CXCL12 (also known as stem cell derived factor-1) and its receptor CXCR4 in mice, wherein the inactivation of the *cxcl12* and *cxcr4* genes resulted in defective hematopoiesis, cardiogenesis, and vascular development (24–26). The biological roles of CXCL12 have also been examined in other vertebrates such as fish and avian species, wherein studies have demonstrated the roles of CXCL12 in muscle formation, vascular development, and homing of hematopoietic stem cells (26–28). While the roles of the amphibian CXCL12 have not been extensively studied, the *X. laevis* CXCL12 has been shown to activate the frog CXCR4 (29).

Here we examine the *X. laevis* peripheral liver as a potential source of precursor cells to GMPs and assess the putative role of CXCL12 in homing of these cells to the myelopoietic bone marrow of this animal.

## Materials and Methods

### Animals, Culture Media and Conditions

Outbred, ~1- to 2-year-old adult *X. laevis* were purchased from Xenopus1 (Dexter, MI), housed, and handled under strict laboratory regulations of Animal Research Facility at the George Washington University (GWU) and as per the GWU Institutional Animal Care and Use Committee regulations (approval number 15-024).

All cell cultures were established in amphibian serum-free medium supplemented with 10% fetal bovine serum, 0.25% *X. laevis* serum, 10 μg/ml gentamycin (Thermo Fisher Scientific), 100 U/ml penicillin, and 100 μg/ml streptomycin (Gibco). Amphibian phosphate-buffered saline (APBS) that was used while isolating the cells has been previously described (18).

### Production of Frog Recombinant Cytokines and Chemokines

*X. laevis* recombinant (r)CSF-1, rEPO, and rCXCL12 were produced using an Sf9 insect cell expression system by a previously described method (18). Briefly, PCR amplicons corresponding to the open reading frames of the respective signal peptide-cleaved proteins were ligated into the pMIB/V5 His A vector. Sf9 insect cells were transfected with the expression constructs (Cellfectin II, Invitrogen), and positive transfectants were selected using 10 μg/ml of blasticidin, their supernatants were screened for recombinant production by western blot against the V5 epitope on the proteins. The cultures expressing rCSF-1, rEPO, or rCXCL12 were scaled up and grown as 500-ml cultures for 5 days, and their supernatants were collected by centrifugation, concentrated against polyethylene glycol flakes (8 kDa) at 4°C, and dialyzed for 2 days against 150 mM sodium phosphate at 4°C. The recombinant proteins were isolated from these concentrated supernatants via Ni-NTA agarose columns (Qiagen), washed with 2 × 10 volumes of high-stringency wash buffer (0.5% tween 20, 20 mM sodium phosphate, 500 mM sodium chloride, and 100 mM imidazole), and 5 × 10 volumes of low-stringency wash buffer (as above, but with 40 mM imidazole). The recombinants were then eluted in 1-ml fractions with 250 mM imidazole. Western blot analysis was performed against the V5 epitopes on rCSF-1, rEPO, and rCXCL12 to confirm the presence of the recombinants. For each protein, the fractions expressing the recombinant were combined and further concentrated against polyethylene glycol flakes (8 KDa) and dialyzed overnight against APBS at 4°C. The protein concentrations were determined by Bradford protein assays (BioRad), and a protease inhibitor cocktail (Halt protease inhibitor cocktail; Thermo Scientific) was added to the purified proteins, which were then aliquoted and stored at −20°C until use. The composition of the recombinant proteins was tested by western blot against the V5 epitope on the protein (Supplementary Figure 1).

The recombinant control (r-ctrl) was produced by transfecting Sf9 cells with an empty pMIB/V5 His A insect expression vector (Invitrogen), collecting the resulting supernatants and processing these using the same methodology as described for rCSF-1/rEPO/rCXCL12.

### Bone Marrow, Peripheral Liver, and Peripheral Blood Leukocyte Isolation and Culture

The liver periphery (LP) cells and bone marrow (BM) cells were isolated by a previously described method (30). Briefly, *X. laevis* femurs and liver tissues were aseptically excised from five individual frogs (*N* = 5) that had been euthanized by tricaine mesylate overdose and cervical dislocation. Femurs were flushed with 10 ml of ice-cold APBS each, and the flushed cells were washed and re-suspended in culture medium.

The peripheral regions of the frog livers were aseptically peeled off using sterile tweezers and passed through 70-μm nylon mesh cell strainers (Fisher). The isolated cells were layered over 51% percoll (Sigma) (49% APBS) and centrifuged at 600 × *g* at 4°C for 20 min to separate out leukocytes from the red blood cells. The leukocytes containing interfaces were collected and washed with ice-cold APBS prior to culture.

Peripheral blood leukocyte (PBL) isolation was performed as follows. Blood was collected from euthanized animals (*N* = 5) by cardiac puncture into medium (containing 1 mg/ml heparin) and processed over 51% percoll, as described above for LP cells, to isolate PBLs.

All cells were enumerated using trypan blue (Invitrogen) live/dead exclusion method.

Toward the analyses of bone marrow chemokine gene expression, the frog femurs were flushed with APBS to remove non-stromal cells and were then repeatedly flushed with Trizol reagent (Invitrogen) to remove stromal/supporting cells. The RNA isolation and cDNA synthesis were carried out as described below.

To assess the effects of monopoietic and erythropoietic stimuli on LP and BM cells, five adult *X. laevis* (*N* = 5) were injected intraperitoneally (ip) with 5 μg of rCSF-1 or rEPO and euthanized 3 days later, and their LP and BM cells were isolated as described above.

### Isolation of RNA and Quantitative Gene Expression Analyses

For all experiments, total RNA from *X. laevis* cells or tissues was isolated using Trizol reagent (Invitrogen) in accordance to the manufacturer's directions. The isolated RNAs (500 ng total) were reverse-transcribed into cDNAs using cDNA qscript supermix (Quanta), according to the manufacturer's instructions.

All quantitative gene expression analyses were performed using the CFX96 Real-Time System and iTaq Universal SYBR Green Supermix (Quanta). The BioRad CFX Manager software was employed for all expression analysis. All gene expression analyses were performed using the delta∧delta CT method, with expression examined relative to the *gapdh* endogenous control. For all experiments, the relative expression was normalized against the lowest observed expression within respective data set. All primers were validated prior to use, and the sequences of all employed primers are listed in Supplementary Table 1.

### Western Blot Analyses of Cellular Transcription Factor Protein Levels

To examine transcription factor expression at the protein level, *X. laevis* LP and BM cells were isolated from three individual frogs (*N* = 3) and lysed in ice-cold radio-immunoprecipitation assay buffer (Thermo Fisher Scientific) in the presence of halt protease and phosphatase inhibitor cocktail (Thermo Fisher Scientific). Protein concentrations of the cell lysates were determined using Bradford protein assays (BioRad), and 30 μg of total protein per sample was resolved by SDS-PAGE and examined by western blot using mouse monoclonal antibodies against Tal1, Egr1, Gfi1, and actin (Santa Cruz) and a secondary goat anti-mouse HRP-conjugated antibody (Thermo Fisher Scientific). Densitometry was performed using ImageJ software. Prior to western blot analyses, protein sequence alignments of mammalian and *X. laevis* Tal1, Egr1, Gfi1, and actin proteins were performed to ensure that the respective epitopes targeted by each of the above antibodies were conserved in the respective *X. laevis* proteins.

### Chemotaxis Assays

Chemotaxis assays were performed using blind well chemotaxis (Boyden) chambers (Neuro Probe), with 100-fold serial dilutions of rCXCL12, concentrations at 10–10^−7^ ng/ml (in culture medium), loaded into the bottom well of these chambers. The wells were overlaid with 13-mm chemotaxis filters (5 μm pore size; Neuro Probe), with addition of 10^4^ LP cells or PBLs in 200-μl volumes of culture medium to the upper chambers. Chemotaxis assays were incubated at 27°C with 5% CO_2_ for 3 h. Subsequently, the cells/medium was removed from the top chambers, and the upper sides of the filters were wiped with cotton swabs. The filters were then stained with Giemsa stain, and the numbers of migrating cells were determined by counting 10 random fields of view per lower side of each filter (×40 objective). For the chemokinesis experiments, both the lower and the upper wells of the chemotaxis chambers were loaded with the most potent chemoattractive concentrations of rCXCL12 (10^−3^ ng/ml), and the assays were performed as above.

For chemotaxis assays using supernatants derived from BM stroma, *X. laevis* femurs were isolated and gently flushed with 10 ml of APBS to remove any non-stromal cells, thus leaving the stroma intact. The femurs were then incubated overnight (16 h) in 1 ml of culture medium each, and the following day the femur-conditioned medium were centrifuged to remove any cells and debris and concentrated tenfold against polyethylene glycol flakes (8 KDa) at 4°C. These BM-conditioned media (BM-med) were serially diluted to 10^−1^ and 10^−2^ and used for chemotaxis assays as described above. Chemokinesis experiments were performed by adding tenfold concentrated BM-med to both upper and lower chemotaxis chambers.

Combined chemotaxis assays of rCXCL12 and BM-conditioned medium were performed by either loading rCXCL12 (10^−3^ ng/ml) into lower chemotaxis chambers and BM-med (tenfold concentrated) into upper chambers or vice-versa, with the target PBLs or LP cells (10^4^) added to upper chambers. The enumerated chemotaxis was compared to cell migration toward the rCXCL12 (10^−3^ ng/ml) or BM-med alone (in lower chambers).

Cells derived from five individual animals (*N* = 5) were used for each and all chemotaxis assays and all assays performed as described above.

### *In vitro* Culture of Peripheral Liver Cells Within Frog Bone Marrow

Frog femurs and LP cells were isolated as described above. The femurs were cut at the condyles on one side of each bone, thereby creating openings into individual femurs. For each animal, one femur bone was then gently flushed with APBS, while the other was flushed with 10 ml of methanol to fix the BM stromal/supporting cells. The isolated LP cells (10^5^) from respective animals were introduced into each of the two femurs from the corresponding animal by gently placing the opening side up into 10 ml of semi-solid culture medium (10% methyl cellulose) and incubating for 3 days at 27°C and 5% CO_2_. Subsequent to this incubation, femurs were cut at the opposite condyles, and the cells were collected by flushing each bone with APBS and enumerated using trypan blue live/dead exclusion method. This experiment was repeated three times, each time using cells from six individual animals (*N* = 6) per experiment.

### Statistical Analysis

For all gene expression and densitometry data, statistical analysis was conducted using paired Student's *T*-test. Chemotaxes data were examined using ANOVA and *post-hoc* Tukey tests via Graphpad Prism 7.0 software. Probability level of *P* < 0.05 was considered significant.

## Results

### *X. laevis* Peripheral Liver and Bone Marrow Cells Possess Distinct Expression Profiles of Lineage-Specific Transcription Factors

**Figure 1:**
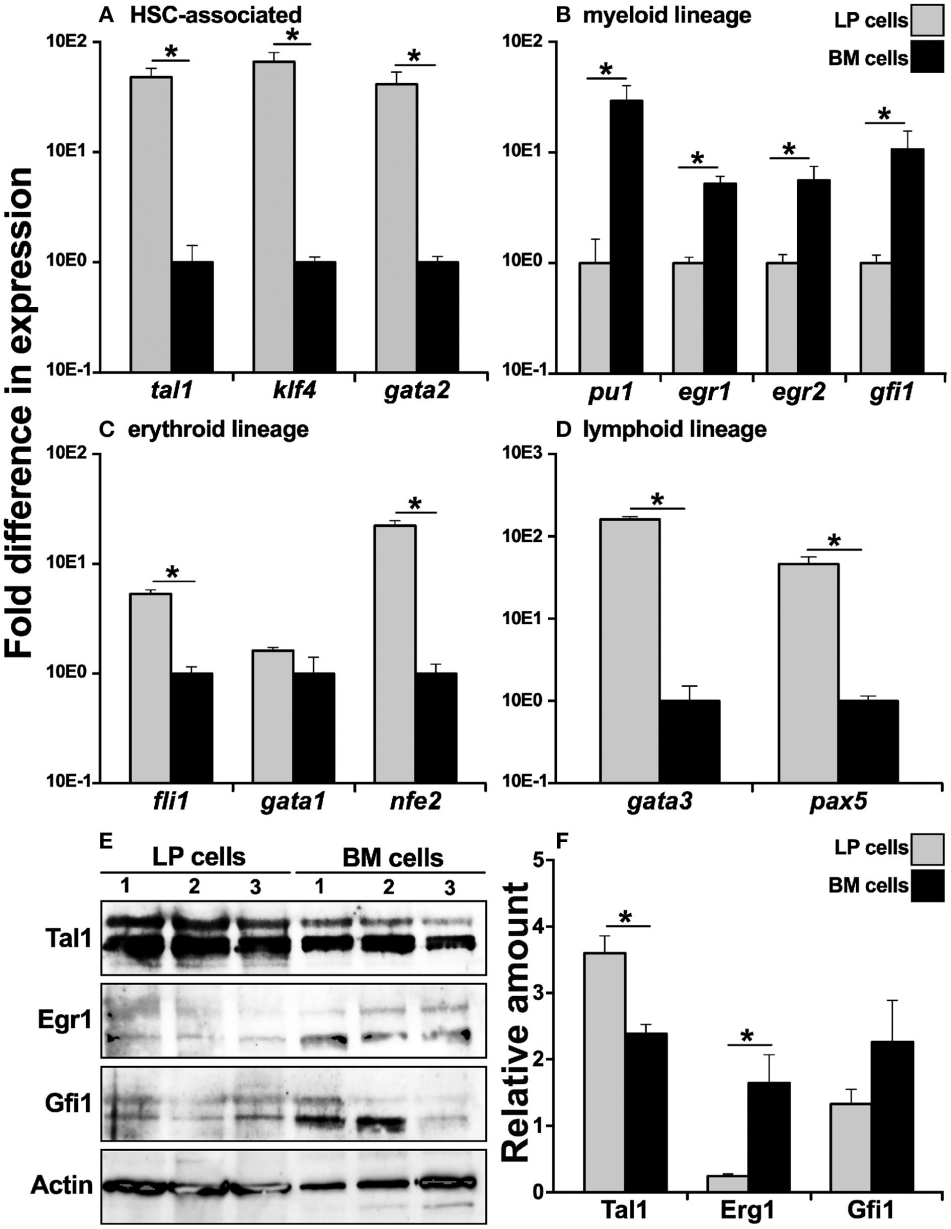
Analysis of lineage-specific transcription factor gene expression in *X. laevis* liver periphery and bone marrow cells. *X. laevis* liver periphery and bone marrow cells were isolated and examined by qPCR for their expression of **(A)** HSC-associated, **(B)** myeloid lineage, **(C)** erythroid-lineage, and **(D)** lymphoid-lineage transcription factor genes. All gene expressions were quantified relative to the *gapdh* endogenous control and normalized against the lowest observed expression. 30 μg of total cell lysate proteins from LP and BM cells was used for western blot analysis to determine the protein levels of **(E)** Tal1, Egr1, and Gfi1 with beta actin as a loading control. The protein levels were quantified by **(F)** densitometry analyses using ImageJ software. Results are means ± SEM (**A–D**: *N* = 5; **E,F**: *N* = 3) and *asterisk* overhead of *horizontal lines* denotes statistical significance, *P* < 0.05.

Since the *X. laevis* peripheral liver is host to most hematopoiesis in this animal (14–16), we examined whether this tissue is the source of cells that commit toward the bone marrow resident granulocyte macrophage precursors (GMPs). To this end, we compared cells from the *X. laevis* LP and BM for their respective gene expression of key transcription factors (TFs) associated with distinct blood cell lineage commitment. In comparison to BM cells, the LP-derived cells exhibited greater mRNA levels for TFs associated with hematopoietic stem cells (HSCs), including *tal1* (31), *klf4* (32), and *gata2* (33), thus corroborating that the peripheral liver is the principal hematopoietic site in *X. laevis* (Figure 1A). Interestingly, myeloid lineage-specific TFs, including *pu1* (34), *egr1* (35), *egr2* (36), and *gfi1* (37), were expressed at significantly greater levels by the BM cells compared to the LP cells (Figure 1B), supporting our previous finding that GMPs reside in the *X. laevis* bone marrow and are absent from their peripheral liver (17, 18). Furthermore, in comparison to BM cells, the LP cells displayed significantly greater gene expression for erythroid lineage TFs, including *fli1*(38) and *nfe2* (39), while *gata1* (40) was expressed at comparable levels in the both cell types (Figure 1C). The lymphoid lineage TFs *gata3* (41) and *pax5* (42) also displayed significantly greater mRNA levels in the LP cells compared to BM cells (Figure 1D). These findings supported our previous observations that the *X. laevis* peripheral liver hosts most blood cell development, excluding the bone marrow-mediated myelopoiesis (17).

To confirm the respective hematopoietic and myelopoietic nature of the *X. laevis* LP and BM cells, we assessed the two cell types for their relative protein levels of myeloid (Gfi1, Egr1) and HSC-associated (Tal1) transcription factors by western blot (Figures 1E,F). As expected, while LP cells possessed significantly greater protein levels of Tal1 (non-phosphorylated, lower bands; phosphorylated, upper bands), the *X. laevis* BM cells exhibited relatively more robust Gfi1 and Egr1 (non-phosphorylated, lower bands; phosphorylated, upper bands) protein levels (significantly so for Erg1) (Figures 1E,F).

### *X. laevis* Bone Marrow and Liver Periphery Cells Respond to Monopoietic and Erythropoietic Stimuli, Respectively

**Figure 2:**
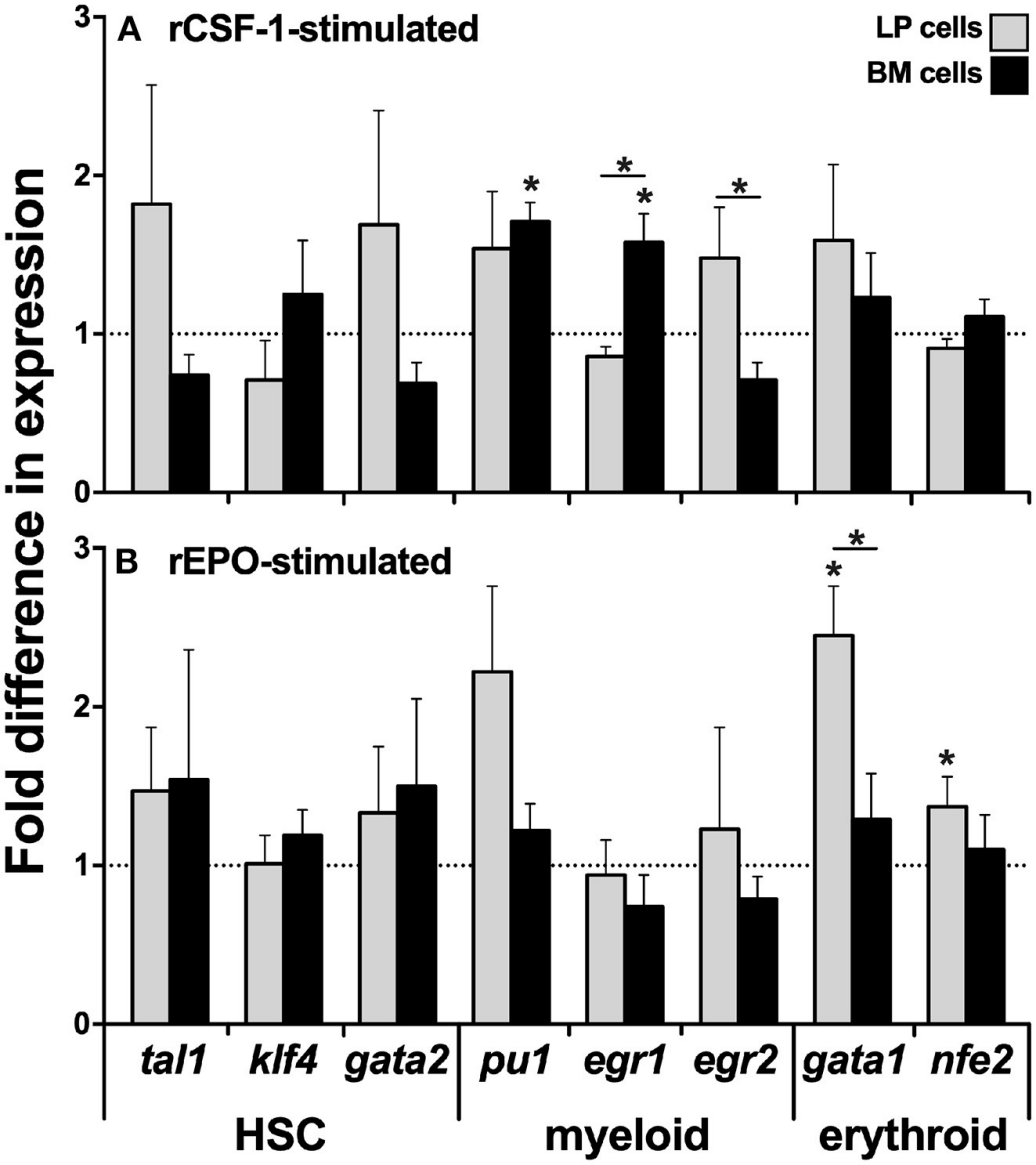
Analysis of lineage-specific transcription factor gene expression in liver periphery and bone marrow cells from rCSF-1- and rEPO-stimulated *X. laevis*. Adult *X. laevis* frogs were injected intraperitoneally with 5 μg of rCSF-1, rEPO, or equal volumes of r-ctrl, and 3 days later their liver periphery and bone marrow cells were assessed for lineage-specific transcription factor gene expression in **(A)** rCSF-1-stimulated and **(B)** rEPO-stimulated cells. The examined genes included hematopoietic-associated TFs: *tal1, klf4, gata2, nfe*2; myeloid-lineage TFs: *pu1, egr1, egr2*; and erythroid lineage TFs: *gata1, nfe2*. All gene expressions was quantified relative to the *gapdh* endogenous control and the gene expression is presented relative to the respective gene expression in *r*-ctrl-treated animals; denoted by *dashed lines*. Results are means ± SEM (*N* = 5) and *asterisk* overhead of *horizontal lines* denote statistical significance between the two cell types and (*) denotes statistical differences between the r-ctrl and respective r-growth factor stimulation, within respective cell types, *P* < 0.05.

Because we observed significantly greater myeloid TF expression by the BM cells, while the erythroid lineage TFs were more robustly expressed by the LP cells (Figures 1B,C, respectively), we next analyzed the expression of these respective lineage-specific markers in LP and BM cells isolated from animals that had been stimulated with recombinant forms of the principal mediators of monopoiesis and erythropoiesis, colony stimulating fator-1 (rCSF-1) or erythropoietin (rEPO), respectively. To this end, frogs were injected intraperitoneally (ip) with rCSF-1 or rEPO, and 3 days later, their LP and BM cells were examined for changes in TF gene expression. Neither LP cells nor BM cells from animals stimulated with either growth factor exhibited significant differences in their gene expression of the HSC-specific *tal1* and *klf4* TFs (Figures 2A,B). Interestingly, LP cells from rEPO-stimulated animals possessed significantly greater transcript levels for the erythroid TFs *gata1* and *nfe2* (Figure 2B). Conversely, BM cells, but not LP cells from rCSF-1-treated animals, exhibited a significantly elevated expression of the myeloid TFs *pu1* and *egr1* (Figure 2A). We did not observe changes in the expression of other examined myeloid lineage or HSC-specific TFs in the LP and BM cells from rCSF-1- and rEPO-stimulated frogs (Figures 2A,B).

### *X. laevis* Bone Marrow Produces Factors That Are Chemoattractive to Peripheral Liver Cells and Blood Leukocytes

**Figure 3:**
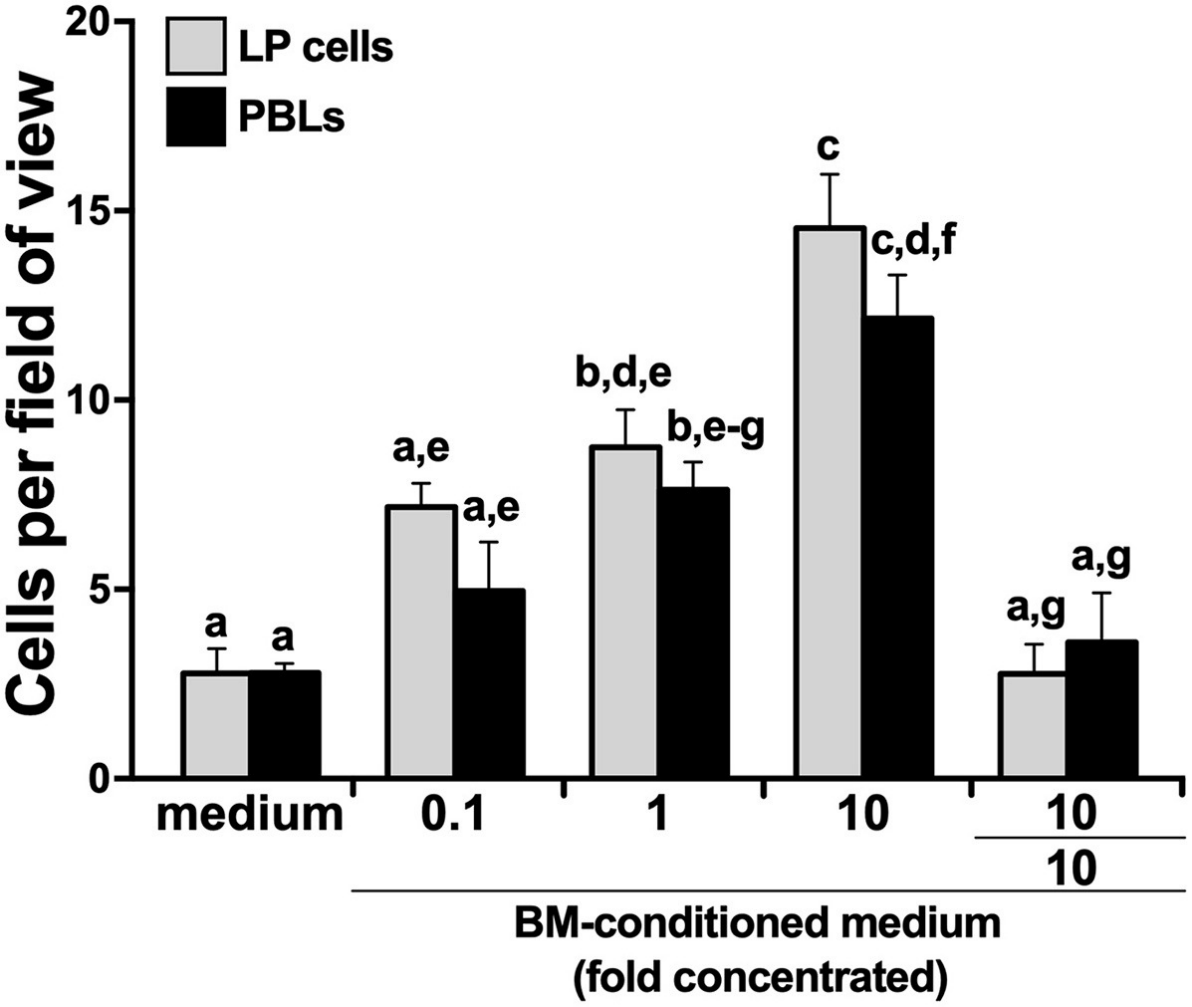
*X. laevis* bone marrow-conditioned medium chemoattracts peripheral liver and blood leukocytes. Tenfold concentrated bone marrow-conditioned medium was serially diluted and examined for its ability to chemo-attract liver periphery (LP) cells and peripheral blood leukocytes (PBLs). Chemokinesis of LP cells and PBLs was measured by adding the tenfold concentrated BM-medium to both the upper and the lower chemotaxis chambers (denoted as 10/10). All chemotaxis/chemokinesis experiments were performed using cells from five individual animals (*N* = 5), enumerating 10 random fields of view per chemotaxis filter per animal. Results are means ± SEM. *Above-head letters* denote statistical designations: experimental groups described by distinct *letters* are statistically different (*P* < 0.05), while those marked by the same letters are not.

We hypothesized that the *X. laevis* myeloid precursors originate in the LP and are traffick through blood circulation to the BM in response to specific BM-produced chemoattractive factors. To test this notion, we opened *X. laevis* femurs, flushed them to remove resident leukocytes, and incubated the resulting femur bones in medium, thereby conditioning the medium with any factors that would be produced by the BM stroma/supportive tissue. We then concentrated the conditioned medium tenfold and assessed the capacity of this conditioned medium to chemoattract LP cells and PBLs. As anticipated, both the LP cells and PBL populations displayed dose-dependent migration toward the BM-conditioned medium (Figure 3). The LP and peripheral blood cells migrating toward the BM-conditioned medium possessed somewhat mixed cytology, reminiscent of immature myeloid-lineage cells (Supplementary Figure 2).

To delineate whether this migration was gradient dependent (chemotaxis) or gradient independent (chemokinesis), we repeated the migration studies, this time adding BM stroma-condition medium (tenfold concentrated) to both upper and lower chemotaxis chambers, thereby disrupting any chemoattractant gradient. This resulted in significantly diminished migration of both LP cells and PBLs (Figure 3), indicating that the factors present in the conditioned medium were eliciting chemotaxis rather than chemokinesis.

### *X. laevis* Peripheral Liver and Bone Marrow Stroma Possess Unique Chemokine Gene Expression Profiles

**Figure 4:**
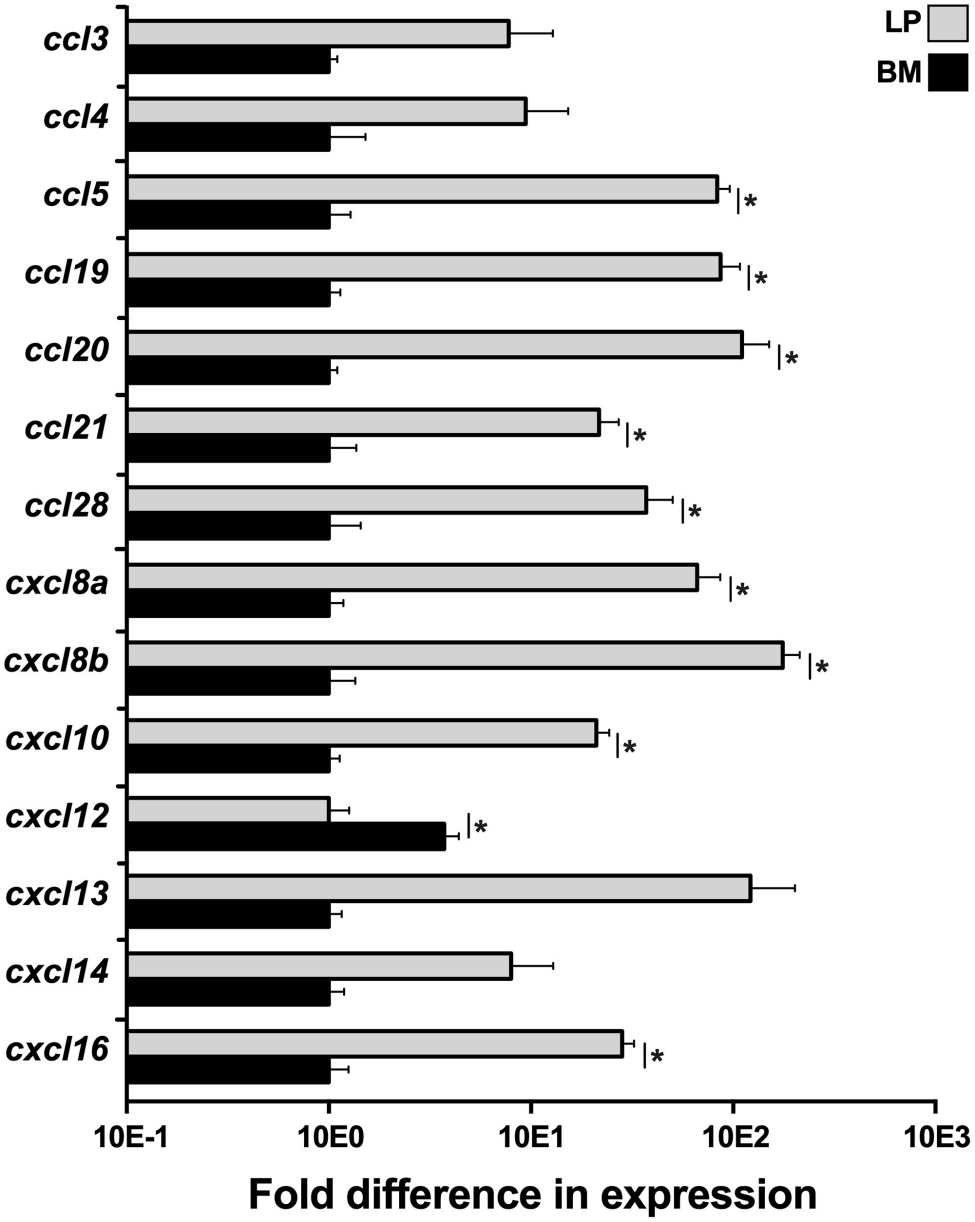
Analysis of chemokine gene expression in *X. laevis* liver periphery and bone marrow. Frog liver periphery (LP) and bone marrow (BM) from five individual animals (*N* = 5) were examined for their expression of a panel of chemokine genes by qPCR. All gene expressions were quantified relative to the *gapdh* endogenous control and normalized against the lowest observed expression. Results are means ± SEM and (*) overhead of *horizontal lines* denote statistical significance, *P* < 0.05.

To elucidate what chemokines might be produced by the *X. laevis* BM stroma, we compared the LP and BM stromal cells for their gene expression of a panel of chemokines, including *ccl3, ccl4, ccl5, ccl19, ccl20, ccl21, ccl28, cxcl8a, cxcl8a, cxcl10, cxcl12, cxcl13, cxcl14*, and *cxcl16* (Figure 4). Most notably, the BM stromal cells possessed significantly greater transcript levels of a single chemokine, *cxcl12*, while the LP cells displayed greater mRNA levels of all other examined chemokines (Figure 4).

### CXCL12 Is Chemotactic to *X. laevis* LP Cells and PBLs

**Figure 5:**
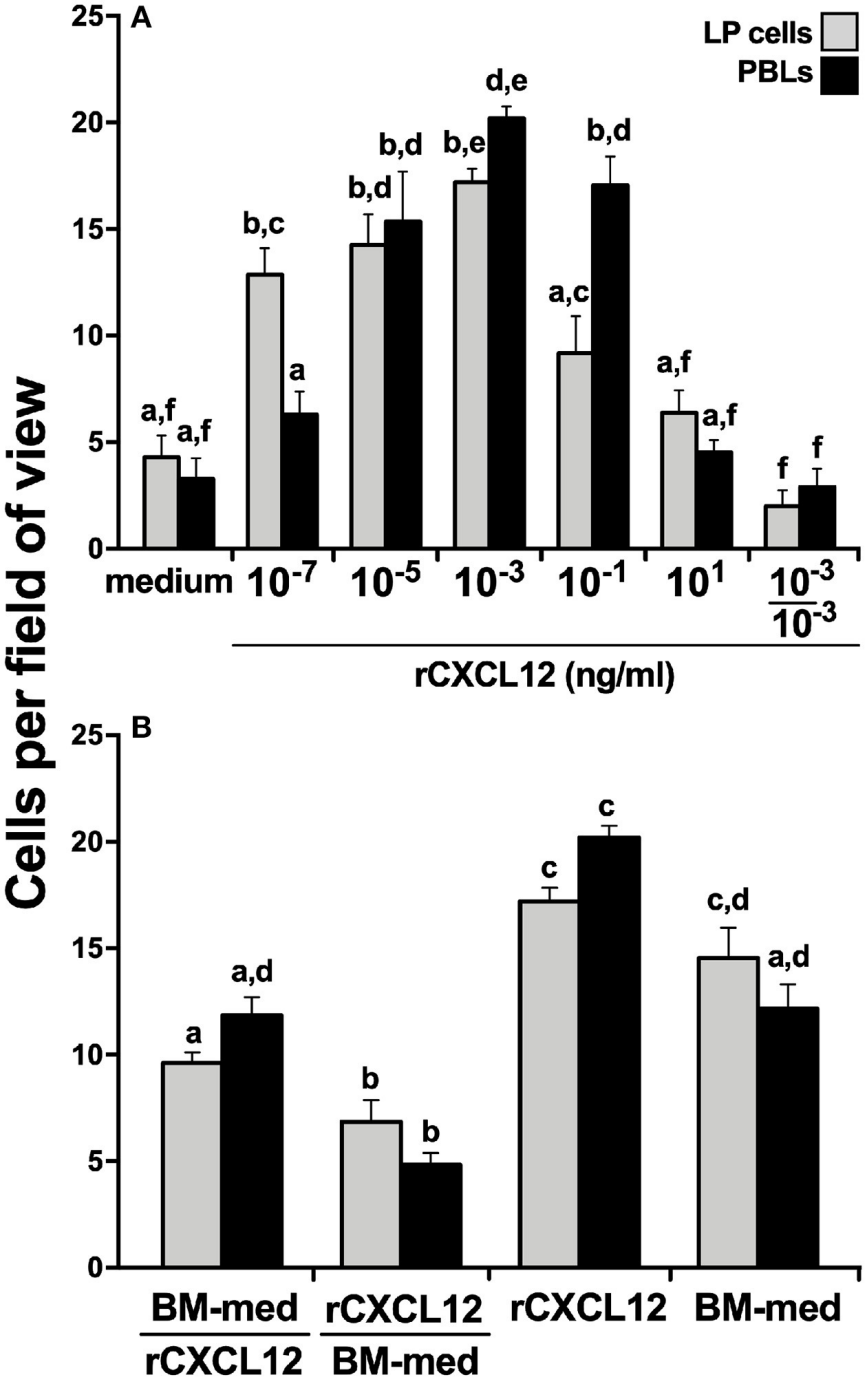
*X. laevis* rCXCL12 is chemotactic to liver periphery and peripheral blood leucocytes. A recombinant *X. laevis* CXCL12 (rCXCL12, 10^−7^-10^1^ ng/ml) was examined for its capacity to chemoattract **(A)** liver periphery (LP) cells or peripheral blood leucocytes (PBLs; 10^4^ cells/well) with chemokinesis assessed by adding 10^−3^ ng/ml of rCXCL12 (optimal dose) to both the upper and the lower chemotaxis chambers. **(B)** The relative capacities of tenfold concentrated bone marrow conditioned medium (BM-med) and rCXCL12 (10^−3^ ng/ml) to chemoattract LP cells and PBLs (10^4^ cells/well) were compared through chemokinesis experiments by adding either BM-med or rCXCL12 and rCXCL12 or BM-med to the upper and lower chemotaxis chambers, respectively (denoted as BM-med/rCXCL12; rCXCL12/BM-med) and compared to BM-med or rCXCL12 in only bottom wells. All chemotaxis/chemokinesis experiments were performed using cells from 5 individual animals (*N* = 5), enumerating 10 random fields of view per chemotaxis filter per animal. Results are means ± SEM. Above-head *letters* denote statistical designations: experimental groups described by distinct letters are statistically different (*P* < 0.05), while those marked by the same letters are not.

To confirm the roles of the CXCL12 in the *X. laevis* homing of myeloid cells, we generated a recombinant form of the *X. laevis* CXCL12 (rCXCL12) and performed chemotaxis assays with LP cells and PBLs. As hypothesized, the rCXCL12 elicited concentration-dependent migration of both LP cells and PBLs (Figure 5A). Notably, at the lowest examined dose of 10^−7^ ng/ml, rCXCL12 resulted in significantly greater migration of LP cells compared to PBLs, whereas significantly greater numbers of PBLs than LP cells were recruited at 10^−1^ ng/ml of the chemokine (Figure 5A). Moreover, our chemokinesis studies indicated that rCXCL12 was eliciting chemotaxis rather than chemokinesis of both LP cells and PBLs (Figure 5A).

Because the *X. laevis* BM harbor GMPs and displayed robust *cxcl12* gene expression (Figure 4), while rCXCL12 was chemotactic to LP cells and PBLs (Figure 5A), we hypothesized that CXCL12 may be a major component of BM-conditioned medium. To address this notion, chemokinesis experiments were carried out using BM-conditioned medium (BM-med, obtained as described above and concentrated tenfold) and rCXCL12, with either rCXCL12 in the bottom chemotaxis chambers and with BM-med loaded into the upper wells, or vice-versa. LP cell and PBL chemotaxis was substantially reduced in either condition (Figure 5B) (significantly so when rCXCL12 was added to top wells), suggesting that indeed CXCL12 may be a major chemotactic component of BM-conditioned medium as it ablates the gradient-dependent chemotaxis elicited by BM-conditioned medium. Both LP cells and PBLs displayed a significantly greater migration toward rCXCL12 than toward the BM-med (Figure 5B), presumably owing to the greater concentration gradient established by the rCXCL12 than present in the BM-med.

### The rCXCL12 Chemoattracts Myeloid-Lineage Cells

**Figure 6:**
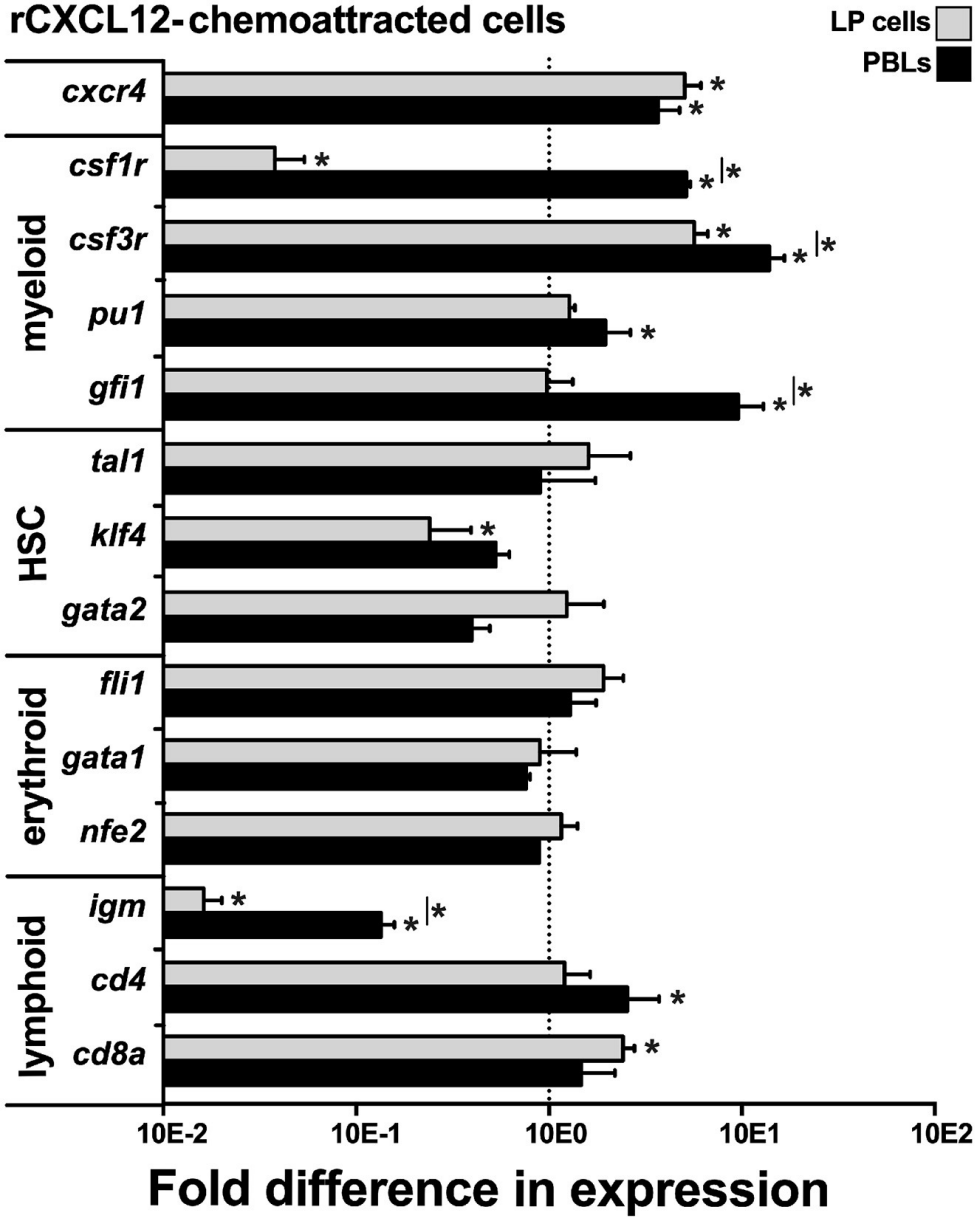
*X. laevis* rCXCL12 chemoattracts myeloid-lineage cells. Chemotaxis assay using the optimal concentration of rCXCL12 (10^−3^ ng/ml) was performed on liver periphery (LP) cells and peripheral blood leucocytes (PBLs; 10^4^ LP cells or PBLs/well, cell from five individual frogs, *N* = 5), and the chemoattracted cells were examined for their gene expression of *crcr4* (receptor for CXCL12); lineage specific markers for myeloid: *csf1r* (macrophage), *csf3r* (granulocyte), *pu1, gfi1*; HSC-associated: *tal1, klf4, gata2*; erythroid: *fli1, gata1, nfe2*, and lymphoid cell populations: *igm* (B cell)*; cd4* (T helper cell); and *cd8* (cytotoxic T cell) by qPCR. All gene expressions was quantified relative to the *gapdh* endogenous control and normalized against the corresponding gene expression observed in the LP cells or PBLs (input, indicated by the *dashed line*) used in these chemotaxis experiments. Results are means ± SEM, (*) denotes statistical differences from the gene expression in total input LP or PBL population (indicated by the *dashed line*) and (*) above *horizontal bars* denote statistical differences between LP cells and PBLs, *P* < 0.05.

To define the lineage commitment of the rCXCL12-responsive LP cells and PBLs, we repeated the rCXCL12 chemotaxis experiment and isolated from the bottom chemotaxis chambers the LP cells and PBLs that migrated toward this chemokine. We then examined these cells for their gene expression of *cxcr4*, the cognate receptor for CXCL12 (43) as well as a panel of myeloid, hematopoietic, erythroid, and lymphoid cell lineage markers (Figure 6). Notably, both the LP cells and PBLs that chemotaxed toward rCXCL12 exhibited robust (compared to total input LP cells and PBLs, respectively) gene expression of CXCR4. While the LP cells chemotaxed toward rCXL12 possessed significantly lower gene expression of the macrophage *csf1r* (*c-fms*) compared to total LP cells; rCXCL12-recruited PBLs possessed significantly greater *csf1r* mRNA levels than total PBLs, and both rCXCL12-recuited LP cells and PBLs possessed significantly greater transcript levels for the granulocyte *csf3r* marker than total LP and PBL cells, respectively (Figure 6). Moreover, the PBLs but not LP cells recruited toward the rCXCL12 exhibited greater expression of myeloid lineage TFs, *pu1*, and *gfi1* than seen in total respective PBL and LP cells (Figure 6).

The rCXCL12-recruited LP cells and PBLs did not exhibit significantly greater levels of any of the examined HSC (*tal1, klf4, gata2*) or erythroid (*fli1, gata1, nfe2*)-lineage TF genes, compared to total respective cell subsets, while the rCXCL12-recruited LP cells possessed significantly lower expression of *klf4* (Figure 6). Compared to total LP cells and PBLs, the rCXCL12-recruited LP cells and PBLs exhibited significantly lower gene expression of *igm* [expressed by B cells; (44)], and while the recruited PBLs possessed greater *cd4* [expressed by T helper cells and some macrophages; (45, 46)] transcript levels, the chemotaxed LP cells exhibited greater mRNA levels of *cd8a* [expressed by cytotoxic T cells and some dendritic cells; (47, 48)] (Figure 6).

### The *X. laevis* Bone Marrow Supports the Survival of Peripheral Liver-Derived Cells

**Figure 7:**
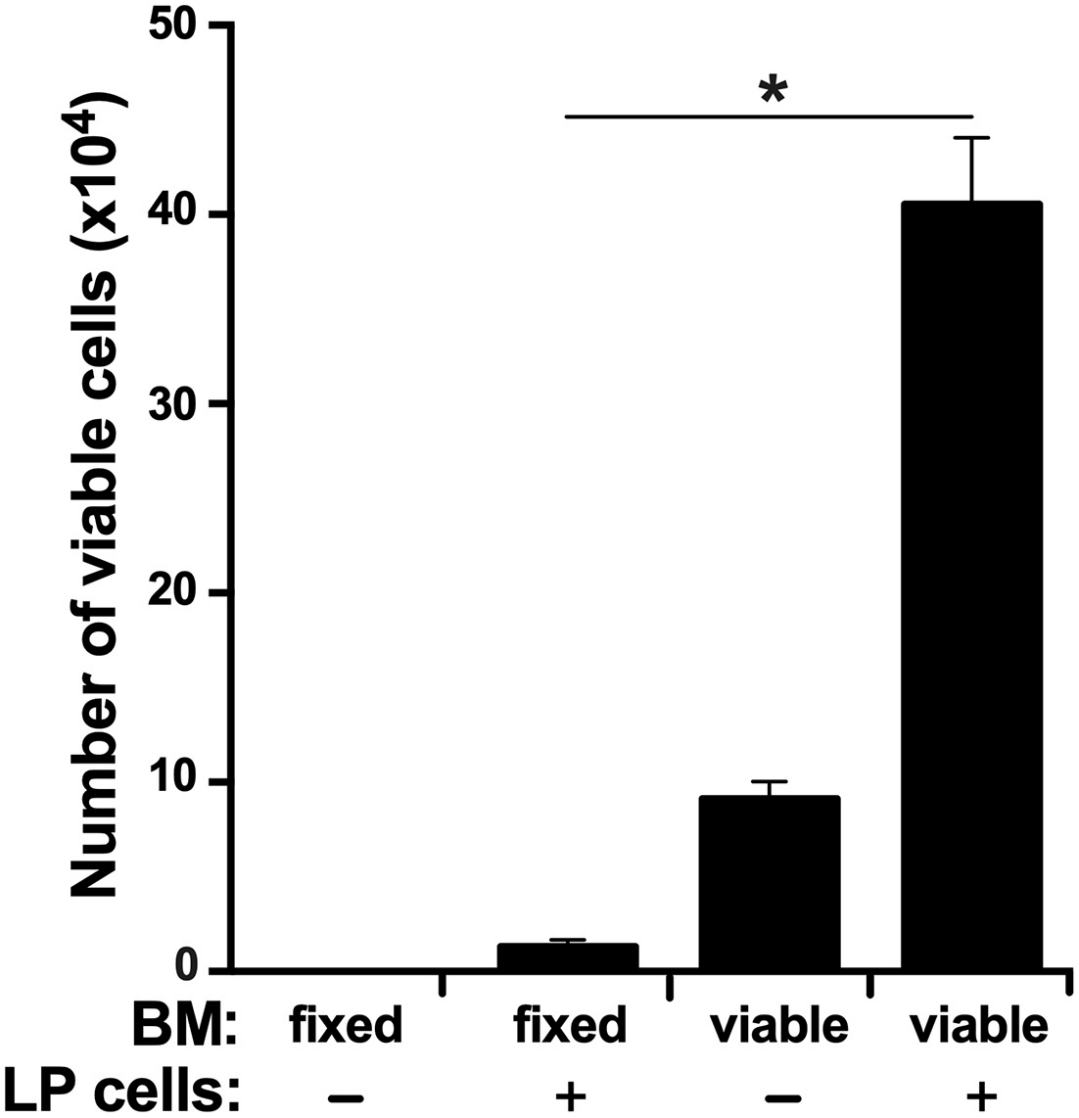
The *X. laevis* bone marrow supports the survival of liver periphery cells. The frog femurs were isolated and cut at the condyles on one side of each bone to create an opening. One femur from each animal was flushed with saline and the other with methanol to fix the stromal/supportive cells. Liver periphery (LP) cells (10^5^ cells per femur from the same respective animals) were introduced into each of the femurs and placed in semi-solid medium with the open-end facing up. After 3 days of incubation, the viable cells in these femurs were enumerated. Results represent combined data derived from three independent such experiments, each experiment assessing tissues/cells from six individual frogs (*N* = 6 per experiment; *N* = 18). Results are means ± SEM and (*) overhead of *horizontal lines* denote statistical significance, *P* < 0.05.

In accordance to the above findings, we reasoned that if the LP is indeed the source of myeloid cell precursors that home to the BM, then the BM should be capable of supporting LP cell survival *in vitro*. To test this idea, we isolated LP cells and cultured them *in vitro* in semi-solid medium within flushed *X. laevis* femurs or femurs with methanol-fixed (and washed) stroma/supportive tissue. As expected, the LP cells displayed significantly greater survival when cultured within viable femur bones as compared to femurs that had been methanol-fixed, thus indicating that the BM is capable of supporting LP cells (Figure 7).

## Discussion

Mammalian hematopoiesis begins in the yolk sac, shifts to the aorta-gonad-mesonephros region of the developing embryo, then to the fetal liver followed by fetal spleen, and ultimately to the bone marrow (49). Similarly, during the early life of avian species, the liver acts as a hematopoietic site, with hematopoiesis later shifting to the avian bone marrow (5, 6). Interestingly, our previous findings indicate that hematopoiesis is segregated between different tissues in the adult *X. laevis*, with myelopoiesis occurring in the bone marrow and to our knowledge, the remaining blood cell development being facilitated by the peripheral liver (14–18). Notably, while the importance of transcription factors to hematopoiesis and lineage commitment has been well-established across vertebrates (19–21), our present transcription factor gene and protein expression analyses of the *X. laevis* peripheral liver and bone marrow cells corroborate our previous findings (17, 18) that while the peripheral liver hosts most hematopoiesis of this animal, the myelopoiesis and GMPs are segregated to the *X. laevis* bone marrow.

Numerous factors contribute to the specialized niche microenvironments within hematopoietic sites to facilitate blood cell development. These include specific cell populations that assist in the interactions between HSCs and the supportive tissues through anchoring/mobilization and production of cytokines/growth factors and chemokines, in addition to other instructive stimuli that may facilitate hematopoietic cell maintenance and regulation (19, 50, 51). Consistent with this, it is possible that one or several of such factors that are crucial for GMP homing, maintenance, and/or differentiation are present in the bone marrow and are absent from the peripheral liver, thus necessitating the segregation of hematopoiesis across these two sites within *X. laevis*. Interestingly, while the blood cell development in adult aquatic anuran amphibian species occur in their peripheral liver tissues, as illustrated in *Xenopodinae* (14–16), this process occurs in the bone marrow of more terrestrial anuran species (52, 53), presumably exemplifying a step-wise evolutionary transition of hematopoiesis from hepatic tissues to bone marrow. Supporting this theory, more recently diverged terrestrial anuran amphibians, such as those of *Rana* genus, utilize their bone marrow as sites for erythropoiesis (54), while in the phylogenetically older aquatic anurans such as *Xenopodinae*, erythropoiesis occurs in the peripheral liver and is absent from their bone marrow (14, 17). Perhaps this suggests that from an evolutionary standpoint, the use of bone marrow as the principal hematopoietic site co-evolved with adaptation of vertebrate life from water to land. This notion is well-corroborated by the fact that most (relatively primordial) aquatic amphibians, such as those of *Gymnophiona* (legless caecilians and species more closely related to them) and *Urodela* (newts and salamanders) orders, are devoid of bone marrow hematopoiesis (52, 55), whereas terrestrial salamanders of the family *Plethodontidae* exclusively utilize their bone marrow toward granulopoiesis and lymphopoiesis (56).

The use of bone marrow as a site of hematopoiesis by amphibians appears to have co-evolved with progressively greater vascularization of this site and coincides with adaptation toward more terrestrial (rather than aquatic) life (57). For example, the bone marrow of the *Triturus pyrrhogaster* newt is composed predominantly of fat cells, with very poor vascular innervation and an apparent lack of any hematopoietic activity (57). Evolutionarily primordial aquatic anurans such as *Bombina* and *Xenopus* possess relatively rudimentary vascularization of their bone marrow compared to mammals and appear to host minimal bone marrow hematopoiesis, which appears to be limited to myelopoiesis in *Xenopus* (57). By contrast, more recently diverged terrestrial amphibians possess bone marrow with considerably more pronounced vascularization that is more akin to that seen within the mammalian bone marrow and coinciding with much greater hematopoiesis taking place within this site (57). As bone marrow-mediated hematopoiesis appears to have co-evolved with greater vascularization of this site, it is reasonable to speculate that this vascularization in turn would facilitate more efficient migration of HSCs to and from this site in response to chemotactic cues such as CXCL12.

Chemokines are not only critical to immune responses but also perform a plethora of functions such as mediating the migration, tissue homing, proliferation, mobilization, and survival of HSCs (22). In turn, during monopoiesis *csf1r* gene expression increases with myeloid lineage commitment (58, 59). Conversely, in addition to myeloid-lineage cells, *csf3r* (granulocyte colony-stimulating factor receptor, *gcsf*) is also expressed by mammalian HSCs, which facilitates CSF-3 (granulocyte colony stimulating factor, G-CSF)-mediated mobilization of HSCs out of the mammalian bone marrow into circulation (60, 61). Notably and compared to total LP cells, the rCXCL12-recruited LP cells possessed lower expression of *csf1r* but greater mRNA levels of *csf3r*. Conversely, the rCXCL12-recruited PBLs exhibited greater transcript levels for both myeloid receptor genes as compared to total PBLs. Concurrently, the CXCL12-recruited PBLs but not LP cells possessed significantly greater gene expression of transcription factors associated with myeloid-lineage commitment. Accordingly, we postulate that in *X. laevis*, CXCL12 (and very likely other factors) mobilizes cell population(s) with GMP potential out of the liver periphery into blood circulation, while the commitment to the GMP lineage occurs in circulation, presumably in response to myeloid growth factors. These GMP progenitor(s) is/are then recruited to the bone marrow by the bone marrow-produced CXCL12. The significantly lower transcript levels of *cxcl12* in the peripheral liver compared to that of the bone marrow presumably facilitates the egress of HSCs with GMP potential, resulting in their migration toward the bone marrow through blood circulation in a CXCL12 concentration gradient-dependent manner. We anticipate that the initial GMP lineage commitment occurs *en route* to the bone marrow and that further lineage-specific differentiation then ensues in the BM in response to local cues and growth factors, giving rise to myeloid cells such as macrophages and granulocytes (17, 18). Indeed the mammalian CXCL12 is essential to the homing of adult HSCs to the bone marrow and is crucial to the migration of HSCs from fetal liver to the bone marrow during development (24, 25). It is thus intriguing that a similar phenomenon appears to facilitate the GMP population of the adult *X. laevis* bone marrow toward myelopoiesis.

HSCs of adult mammals are known to migrate predominantly toward a CXCL12 concentration gradient (62), while the activation of the HSC cell surface-expressed CXCR4 by CXCL12 is indispensable to the regulation of HSC migration during adult life (24). In fact, targeted deletion of CXCR4 results in decreased HSC pools in the mammalian bone marrow (24). Although the *X. laevis* CXCL12 has been shown to signal through CXCR4 (23), the precise role(s) of this chemokine in *X. laevis* hematopoiesis in general and myelopoiesis in particular remain to be fully defined. Notably, our present results indicate that the *X. laevis* CXCL12 is more prominently expressed by their bone marrow than their hematopoietic peripheral liver and appears to be important to the migration/homing of some sort of GMP population(s) from the liver periphery to the bone marrow. It is interesting to consider that in *X. laevis*, CXCL12 has evolved to mediate the migration/homing of one or few hematopoietic progenitor subsets to the bone marrow, rather than functioning (as in mammals) as a more global regulator of HSCs within their peripheral liver, which serves as their principal hematopoietic tissue (14–16). Further studies of the roles of CXCL12 across phylogenetically disparate vertebrates possessing distinct hematopoiesis strategies may elucidate what aspects of the *X. laevis* (and mammalian) bone marrow physiology dictate the use of CXCL12 toward homing of progenitor cells toward this site.

In addition to CXCL12-mediated chemotaxes, the migration of mammalian fetal hepatic HSCs to the bone marrow is influenced by a number of other factors. For example, the role of CXCL12-CXCR4 in the migration of HSCs is augmented by other soluble mediators including stem cell factor, whose chemotactic activity toward mouse fetal hepatic HSCs is synergistic with that of CXCL12 (63). Similarly, activation/signaling through the roundabout guidance receptor 4 (ROBO4) that is expressed by mammalian HSCs aids in the early migration of HSCs from fetal liver to fetal bone marrow and augments the CXCR4-mediated homing and population of HSCs into the adult bone marrow (64). Additionally, adhesion molecules such as cadherins, integrins, and selectins also play important roles in the movement of HSCs to distinct niches. For instance, N-cadherin and integrin β1 expressed on HSCs are involved in the homing and maintenance of these cells in the bone marrow (65, 66). Concurrently, P-selectins and E-selectins expressed by vascular endothelia in the bone marrow promote this HSC migration and homing (67). Furthermore, the migration of HSCs during development is also influenced by extracellular calcium concentration in the bone marrow, which are sensed by the HSC-expressed calcium-sensing receptors (CaRs) (68). Akin to mammals, the recruitment of *X. laevis* GMP into their bone marrow and retention therein undoubtedly depends on a plethora of other factors in addition to CXCL12. Our result showing that the *X. laevis* bone marrow promotes the survival of liver periphery-derived hematopoietic cells supports the notion that in addition to chemokine homing, other factors likely contribute to progenitor cell-bone marrow interactions.

Phylogenetically diverged vertebrate groups possess distinct hematopoiesis strategies, presumably reflecting the physiologies and habitats of those organisms. Despite these differences, vertebrates rely on many of the same soluble mediators, such as growth factors and chemokines, to facilitate their respective blood cell development. We believe that greater understanding of the biological roles of such evolutionarily conserved mediators in the contexts of disparate animal hematopoiesis strategies will grant much clearer understanding of the evolution of vertebrate hematopoiesis.

## Data Availability Statement

The datasets generated for this study are available on request to the corresponding author.

## Ethics Statement

The animal study was reviewed and approved by the Institutional Animal Care and Use Committee (IACUC) (approval number 15-024).

## Author Contributions

AY and LG designed and planned the studies. AY and PR performed the experiments. AY analyzed the data, wrote the manuscript, and prepared the figures. LG contributed to investigation, review, and editing of the manuscript.

### Conflict of Interest

The authors declare that the research was conducted in the absence of any commercial or financial relationships that could be construed as a potential conflict of interest.
