# Supplementary material for: Myelopoiesis of the Amphibian Xenopus laevis Is Segregated to the Bone Marrow, Away From Their Hematopoietic Peripheral Liver
Source: Front Immunol. 2020 Jan 22;10:3015. doi: 10.3389/fimmu.2019.03015 (PMC6987381; doi:10.3389/fimmu.2019.03015)
Supplement: Supplementary file 1 [file Data_Sheet_1.pdf]

Supplementary Material

Supplementary table 1: List of primer sequences

Oligo	Sequence 5'→3'	Oligo	Sequence 5'→3'
*ccl3*_F<br>*ccl3*_R	AGTGTGGACCAGGAGGTAA<br>CTGCTGGTGATATAATAGTCCTTGA	*cxcl16*_F<br>*cxcl16*_R	GTGGGTCCAGGAACTGATATG<br>GCTCAGAGCCCTTGGAATAA
*ccl4*_F<br>*ccl4*_R	TCCAACCCTGGCGTTATATTC<br>GCATTCTTGTCCAGCTTTGTC	*cxcr4*_F<br>*cxcr4*_R	CCAGGACCATGACAGACAAATA<br>CCATCCAATAGCAGCATCCA
*ccl5*_F<br>*ccl5*_R	GTTCTTGTCACCCGGAAGAA<br>TGTGTTGCTTCAGGCATCT	*egr1*_F<br>*egr1*_R	ACCAACAGCAGTAATACCCTATG<br>GATGAGGACGGTGAAGATGAAG
*ccl19*_F<br>*ccl19*_R	ACCATCAACAAGCCGATTCC<br>GAGCTGTATGTTTCTCCGAGTG	*egr2*_F<br>*egr2*_R	TCTCCTACTCGTCCAACTACC<br>CGCTCACTAGATTGAAGATCCC
*ccl20*_F<br>*ccl20*_R	CTCCTGGCTGCTTTGATGT<br>GGATGAAGAATACGGTCTGTGTAT	*fli1*_F<br>*fli1*_R	CTCAGCCAGATCCATACCAAATA<br>GTCAGATAGCAACTCCAAGAGAA
*ccl21*_F<br>*ccl21*_R	ATCCAGTGGCTGCTCAATC<br>CTGGCAATCATAGTCTGTACCC	*gapdh*_F<br>*gapdh*_R	ATGTGTCCGTTGTGGATCTG<br>GATTCCTTTCATTGGTCCCTCT
*ccl28*_F<br>*ccl28*_R	GCTCTCTGTGTCAATCCCAATA<br>CCGGTGTTTCTTTCTTCCCT	*gata1*_F<br>*gata1*_R	CATTGTGGAGGAGGGATATGAG<br>GACAATCAGGCGTTTCTTTGG
*cd4*_F<br>*cd4*_R	GTCGCCCTCCACTAATCTAATC<br>ACCGTTATCCCACGTTTATCC	*gata2*_F<br>*gata2*_R	GTAACGCCTGTGGTCTCTATC<br>GGTAGTTGAGGTCTGACAGTTT
*cd8a*_F<br>*cd8a*_R	GGCATTCACATTCACCCTAAAC<br>TAGCCCGGGACTGATAGAAA	*gata3*_F<br>*gata3*_R	ATCCCTGGCTACAGGACAT<br>GTGGAACCAGAGGTGGAAATAG
*csf1r*_F<br>*csf1r*_R	GAGCAAGGGCACTGATAGTT<br>AAAGGTCTACGGGCAAGATG	*gfi1*_F<br>*gfi1*_R	TCTCTCCGGTCTCCTCAAATC<br>GGTCTCCAGAAATCCTCACATTC
*csf3r*_F<br>*csf3r*_R	TGGATGAAGGACTACAGCTAATG<br>GCCTGTCATCTGTGAGGTTTA	*igm*_F<br>*igm_R*	AACGTTGCCTCTGCAGTCTG<br>TTCTTCAACTCTGACACCTTC
*cxcl8a*_F<br>*cxcl8a*_R	CATAAAGACAGAAAGCAAGCCT<br>GGCTCCAAGCAGATATCG	*klf4*_F<br>*klf4*_R	CAGGTTGCGGAAAGACCTATAC<br>AATTTCCACCCACACCCTTC
cxcl8b_F<br>*cxcl8b*_R	ACGACCCACTCTGCTTTC<br>TTTCTACCCAGCGCTGAG	*nfe2*_F<br>*nfe2*_R	CACACTGACTGATACCCAGATG<br>CCCTTTCTAGACTGGCGATATTT
*cxcl10*_F<br>*cxcl10*_R	GGCTGTGATGAAGTTGAAGTTG<br>GTAGGGCAGTCATGAGTTTGT	*pax5*_F<br>*pax5*_R	CTACGGGTTAGTCATGGATGTG<br>GCAACCTTTGGCTTTGATCC
*cxcl12*_F<br>*cxcl12*_R	ATAAAAAACACATTCGTTCCT<br>ACAGCGGTTAACCACTTGG	*pu1*_F<br>*pu1*_R	TGAATACTACCCGTACCTCAGT<br>AATCAGGGAATCCTTCCAGTTC
*cxcl13*_F<br>*cxcl13*_R	AAGCTGACACGGGTTGAA<br>CAGCTTGTGGATTGACACATAC	*tal1*_F<br>*tal1*_R	CCACAGCTTCTCTCCCTTTAC<br>GTGTCTGGGTCACCAAAGTAG
*cxcl14*_F<br>*cxcl14*_R	GGACTTGTACTGCCGTTCTT<br>GCGTACCTTCCCATGTAATGA		

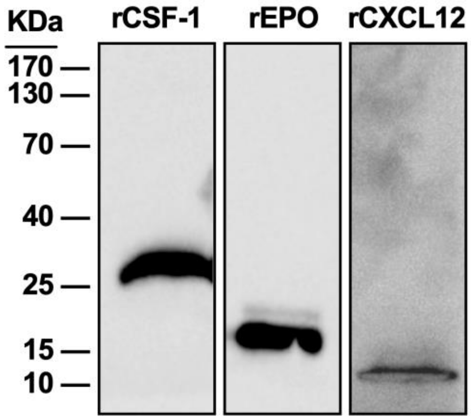

Supplementary Figure 1: Western blot analyses of recombinant proteins colony stimulating factor-1 (rCSF-1), erythropoietin (r-EPO) and chemokine CXC12 (rCXCL12). The recombinant proteins were produced using an insect expression system and isolated via Ni-NTA agarose column purification. The purity of the recombinants was tested by western blot against the V5 epitope on the proteins.

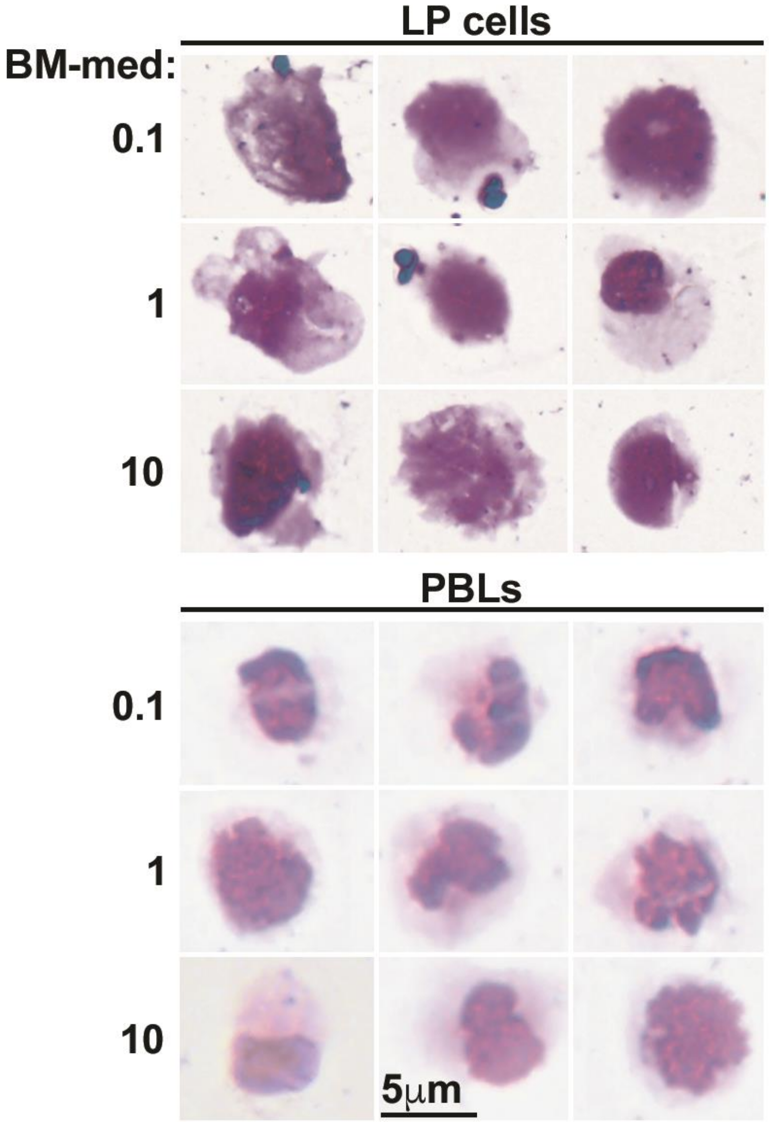

Supplementary Figure 2: *X. laevis* bone marrow-conditioned medium chemoattracts peripheral liver cells and blood leukocytes. Ten-fold concentrated bone marrow-conditioned medium (BM-med) was serially diluted (10, 1, 0.1) and examined for its ability to chemoattract liver periphery (LP) cells and peripheral blood leukocytes (PBLs). The chemoattracted cells from each concentration of BM-med were cytologically examined following Giemsa stain.
